# Web- and Mindfulness-Based Intervention to Prevent Chronic Pain After Cardiac Surgery: Protocol for a Pilot Randomized Controlled Trial

**DOI:** 10.2196/30951

**Published:** 2021-08-30

**Authors:** Geraldine Martorella, Adam W Hanley, Scott M Pickett, Céline Gelinas

**Affiliations:** 1 Tallahassee Memorial Healthcare Center for Research and Evidence-Based Practice College of Nursing Florida State University Tallahassee, FL United States; 2 Center on Mindfulness and Integrative Health Intervention Development College of Social Work University of Utah Salt Lake City, UT United States; 3 Department of Behavioral Sciences and Social Medicine, Center for Translational Behavioral Science College of Medicine Florida State University Tallahassee, FL United States; 4 Ingram School of Nursing McGill University Montreal, QC Canada; 5 Centre for Nursing Research Lady Davis Institute Jewish General Hospital Montreal, QC Canada

**Keywords:** postoperative pain, cardiac surgery, chronic pain, web-based, mindfulness, mobile phone

## Abstract

**Background:**

Cardiac surgery is a frequently performed procedure. However, pain after cardiac surgery may become chronic (lasting >3 months) in adults. Once discharged from the hospital, patients are at greater risk of developing chronic postsurgical pain (CPSP) and of prolonged opioid use, as they need to self-manage their pain. Psychological risk and protective factors such as pain-related catastrophic thoughts and pain acceptance determine their ability to cope and their use of opioids, which is crucial for self-management of pain. Studies on mindfulness-based cognitive therapy (MBCT) have multiplied their potential effects on pain acceptance and catastrophic thoughts. However, web-based MBCT for the prevention of CPSP has not yet been examined.

**Objective:**

The aim of this study is to pilot test a 4-week-long web-based MBCT intervention for adults following discharge from the hospital by assessing the acceptability or feasibility of the intervention and examining preliminary effects on pain intensity, pain interference with activities and opioid use, and pain acceptance and catastrophic thoughts in the 6 months following surgery.

**Methods:**

A double-blinded pilot randomized controlled trial will be used to assess a web-based MBCT intervention. Patients will be selected according to the following criteria: age ≥18 years; first-time elective cardiac surgery via a median sternotomy; worst pain in the past week score ≥4/10; ability to understand and complete questionnaires in English; and ability to use an electronic device such as a smartphone, computer, or tablet. After baseline measures, 32 participants will be randomized into two groups: one receiving both the brief, 4-week-long web-based MBCT intervention and usual care (experimental group) and the other receiving only one standardized, web-based educational session with weekly reminders and usual care (attention control group). Peer-reviewed competitive funding was received from Florida State University’s Council on Research & Creativity in January 2021, as well as research ethics approval from Florida State University's institutional review board.

**Results:**

Recruitment began in June 2021. Unfortunately, because of the current COVID-19 pandemic, recruitment is not progressing as expected. Recruitment strategies are constantly monitored and updated according to latest data and restrictions surrounding the pandemic.

**Conclusions:**

This research is significant because it targets the trajectory of CPSP, a leading cause of disability and opioid misuse. This is the first study to assess MBCT for the prevention of CPSP after cardiac surgery in the recovery phase. This approach is innovative because it promotes self-management of pain through the modulation of individual factors. If successful, the intervention could be expanded to numerous populations at risk of chronic pain.

**International Registered Report Identifier (IRRID):**

DERR1-10.2196/30951

## Introduction

### Background

Cardiac surgery is a common life-saving procedure [1] that can lead to several complications during recovery. One of them is persistent postoperative pain (ie, pain developing after surgery and lasting at least 3 months with other causes of pain excluded [2]), and its incidence seems to have increased [3]. Estimates suggest that up to 37% of patients undergoing cardiac surgery have persistent pain and will potentially develop chronic postsurgical pain (CPSP; ie, pain lasting for more than 3 months following surgery), with 50% of them reporting moderate to severe postoperative pain [3] that compromises their recovery and daily functioning [3-6]. Furthermore, 10% of the patients will develop new prolonged opioid use (ie, opioid use for more than 3 months) [7], which portends addiction and overdose death [8]. Both chronic pain and chronic opioid use can exacerbate heart disease [9-14]. Compounding this human cost, CPSP is expensive, incurring annual direct and indirect costs of US $41,000 per patient in the United States [15].

Various biopsychosocial factors have been found to increase a cardiac surgery patient’s risk of transitioning from acute pain to CPSP [3,5,16,17]. Modifiable psychosocial factors such as pain catastrophizing (ie, an exaggerated negative mental set brought to bear during actual or anticipated painful experience [18]) and pain acceptance (ie, a person’s engagement to take valuable actions despite the pain [19]) have gained much attention from researchers. Indeed, greater levels of catastrophic thinking are associated with higher perceived pain intensity, and greater levels of pain acceptance are associated with better functioning [20,21]. A behavioral intervention targeting known risk factors during the sensitive, subacute recovery period may prevent CPSP development [16,22,23]. With reduced hospital length of stay after cardiac surgery [24,25], patients are at risk for CPSP and prolonged opioid use [26,27] as they become more isolated with relatively elevated levels of pain after discharge [3,28,29]. Moreover, rarely aware of the risk of CPSP [30], patients may seek pain management support only after their pain has become chronic and disability has already surfaced [31]. Teaching patients undergoing cardiac surgery effective, psychological pain self-management strategies that address common CPSP risk (eg, pain catastrophizing) and protective (eg, pain acceptance) factors is likely to improve their pain coping and decrease their opioid use [26,32-34]. Indeed, pain acceptance and catastrophizing have been shown to be theoretically related and mediate the relationship between pain and clinical outcomes [20,35,36]. The subacute phase following discharge from the hospital may be an optimal time for such an intervention, but there is limited research examining the efficacy of psychological interventions for pain self-management in the subacute phase [34,37]. The evidence comes from rather demanding interventions (eg, 60-minute weekly group sessions for a minimum of 8 weeks with additional homework), whose efficacy may be decreased because of limited patient adherence [34]. Therefore, it is critical to translate knowledge on pain-related psychological risk factors into timely interventions promoting pain self-management in this decisive phase of the postsurgical pain trajectory [23,38-41].

Traditionally, interventions based on cognitive behavioral therapy have been used to treat chronic pain, as considerable evidence suggests that they have a modest effect on pain and may also decrease pain-related disability and depression [42,43]. However, more recently, the number of studies on mindfulness-based interventions (MBIs) for chronic pain has increased tremendously [44-46]. MBIs train patients to engage in self-regulation of attention by increasing their awareness and acceptance of present moment thoughts, feelings, and physical sensations [47]. Some research suggests that MBIs induce adaptive neurobiological changes and that people who practice mindfulness have better pain tolerance (ie, “the maximum intensity of a pain-producing stimulus that a subject is willing to accept in a given situation” [48]) than those who do not [49]. The first wave of MBIs for chronic pain, such as mindfulness-based stress reduction, mindfulness-based cognitive therapy (MBCT), or mindfulness-oriented recovery enhancement, largely consist of eight weekly, 2-hour group therapy sessions [21]. However, more recent MBIs for acute and chronic pain have adopted briefer formats, and preliminary evidence suggests that they may be efficacious as well [50,51]. Although more research is needed regarding their feasibility and efficacy across different settings, brief MBIs have already been shown to (1) decrease hospitalized patients’ pain intensity, pain unpleasantness, and anxiety [52]; (2) decrease surgical patients’ preoperative pain intensity, pain unpleasantness, pain medication desire, and anxiety, as well as improve their physical functioning 6 weeks after surgery [53]; and (3) decrease pain intensity, pain unpleasantness, and opioid use in the first month after surgery [54]. In addition, the benefits of MBIs appear to extend beyond pain. MBIs have also been found to improve stress, depressed mood, and sleep quality [55-57], which are all intertwined with chronic pain. Moreover, MBIs are emerging as promising approaches for mitigating heart disease and promoting cardiac rehabilitation [58-60]. Specifically, MBCT—which combines mindfulness strategies and cognitive therapy—is one of the most widely researched MBIs and may be uniquely suited for pain management given its potential effect on pain-related psychological factors such as catastrophic thinking and pain acceptance [21,61-65].

Our preliminary work with a web- and cognitive behavioral therapy–based intervention in the acute or hospitalization phase after cardiac surgery showed that (1) a brief targeted intervention may be able to modulate modifiable psychological risk factors and reduce postoperative pain interference with activities [66,67], (2) patients express the need for additional pain management support after hospital discharge [39], and (3) clinicians recommend interventions that enhance patients’ engagement in their recovery [38]. However, offering additional support and engaging patients in their care can be challenging in terms of time and resources for clinicians and in terms of accessibility for patients. Nevertheless, eHealth approaches seem promising and have the potential to increase active involvement in one’s health [68]. Moreover, they seem to lead to similar or improved perioperative clinical outcomes compared with face-to-face interventions [69]. To our knowledge [70], our intervention is the only web-based intervention designed to prevent CPSP in patients undergoing cardiac surgery. Furthermore, brief web-based MBCT for pain management or prevention of CPSP has not been examined.

### Objectives

In this study, we will pilot test and evaluate a brief 4-week web-based MBCT intervention designed to promote postoperative pain self-management in patients undergoing cardiac surgery during the subacute phase. Guided by a biopsychosocial framework of pain, our team will accomplish two aims using a pilot randomized controlled trial (RCT) in this stage 1 study [71]: (1) to assess the acceptability and feasibility of the brief web-based MBCT intervention for pain self-management in the subacute phase and (2) to examine the preliminary effects of brief web-based MBCT intervention on pain intensity, pain-related interference with physical and emotional function and opioid use (ie, clinical outcomes), and pain-related catastrophic thinking and pain acceptance (ie, therapeutic mechanisms).

## Methods

### Theoretical Framework

**Figure 1:**
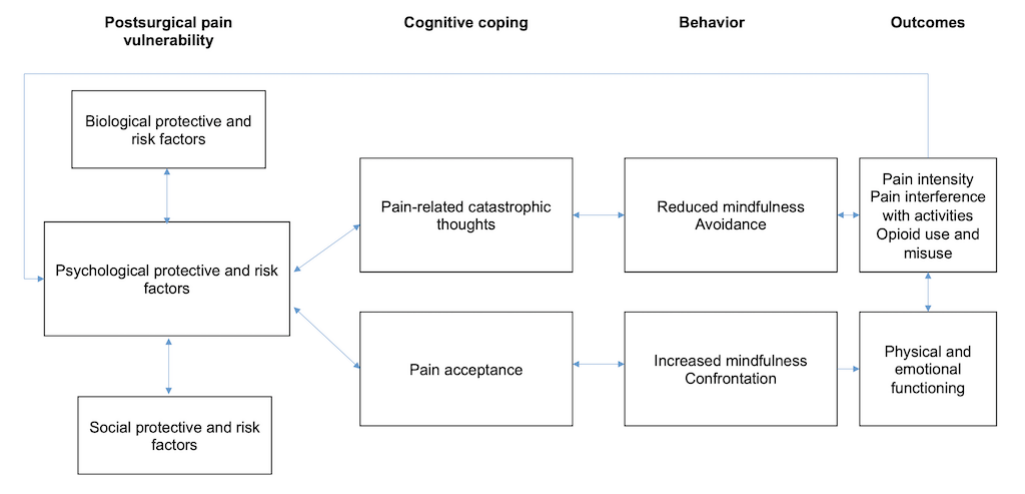
Conceptual model based on the biopsychosocial model of chronic pain.

The biopsychosocial model of chronic pain is the framework of this intervention and study, as it is the most comprehensive foundation for understanding, preventing, and treating chronic pain [72,73]. Psychological risk and protective factors are the cornerstone of this model [72,74-76], as they are relatively modifiable factors involved in pain processes and determine individualized pain reactions, including pain self-management behaviors [77]. Among these factors, pain acceptance and pain-related catastrophic thoughts have been recognized for their contribution to CPSP [34] and have been identified as potential contributing factors to the therapeutic mechanism of MBCT [21,61,62]. For these reasons, these two variables are the targets of the intervention (Figure 1).

### Study Design

A double-blinded pilot RCT will be used to assess the brief web-based MBCT intervention in the 6 months following cardiac surgery (coronary artery bypass grafting and valve replacement). This study received ethical approval from the institutional review board of Florida State University in January 2021. An experienced research assistant (RA) will be responsible for participants’ recruitment and informed consent procedures at the time of follow-up with the surgeon (usually 2-4 weeks after surgery). The study will be advertised in the surgeon’s offices and surgical units with posters and flyers. Social media platforms and newspapers will also be used. If interested, potential participants will contact the RA via telephone or email, and the inclusion criteria will be assessed. After baseline measures have been collected, participants will be randomized into two groups by the principal investigator (PI): one receiving both the brief 4-week web-based MBCT intervention and the usual care procedure (experimental group [EG]); the second group receiving only one standardized educational web-based session in the first week along with weekly reminders for 3 weeks and the usual care procedure (attention control group [ACG]). At the end of the intervention, participants will be asked if they received the intervention to evaluate their blinding. Participants from the ACG will be given the opportunity to receive the entire intervention once the study is complete.

Computer software will generate the randomization schedule: permuted block randomization with block sizes of 4 and an allocation ratio of 1:1. The randomization list and opaque sealed envelopes numbered sequentially will be prepared by the PI’s colleague who will not be involved in this study. The RA who will be responsible for the entire data collection will be blinded to the patient group assignment. Participants in both groups will receive gift cards of US $25 at each of the four data collection time points (baseline, after intervention, 3 months after surgery, and 6 months after surgery) for a total of US $100.

### Settings and Participants

To be able to provide precise estimates of mean and variance that will aid in the planning of a larger and sufficiently powered efficacy trial, a reasonable rule of thumb for continuous variables (eg, pain intensity) is a sample size of 12 per group [78]. Based on previous experience with this population and intervention, we could expect an attrition rate of 20%. However, an attrition rate of 30% could be anticipated in the long term at the 6-month follow-up [43]. Hence, we will recruit 32 participants—16 (50%) per group. Patients will be selected according to the following criteria: (1) age ≥18 years, (2) first-time elective coronary artery bypass grafting and valve replacement via a median sternotomy, (3) worst pain in the past week score ≥4/10 [79], (4) ability to understand and complete questionnaires in English, and (5) ability to use an electronic device such as a smartphone, computer, or tablet. Patients will not be eligible for the study if they (1) had undergone a previous thoracotomy or mastectomy and (2) were unable to consent because of physical or cognitive incapacity.

### Procedures

**Figure 2:**
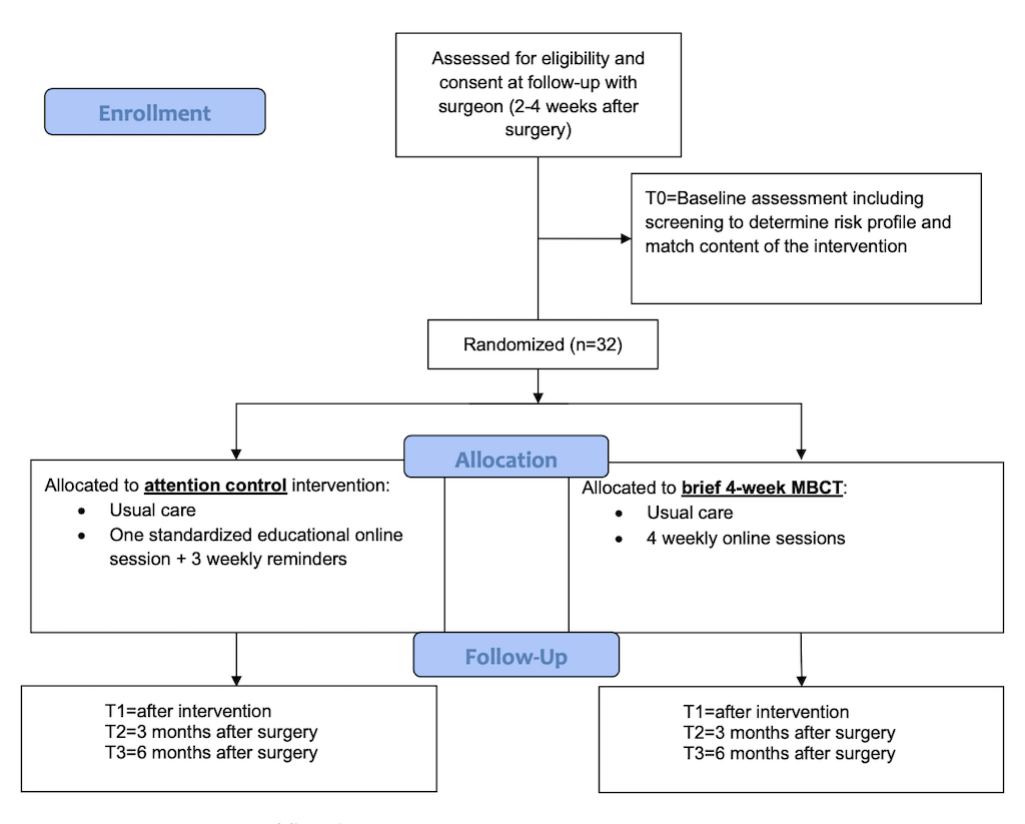
Participant flow diagram. MBCT: mindfulness-based cognitive therapy.

All participants will complete baseline measures via a telephone interview (with responses entered in a Qualtrics survey) at the time of enrollment (T0). Figure 2 depicts participant flow during the study protocol.

### Choice of Comparators

Most MBI studies include the usual care control groups. A meta-analysis conducted by the Agency for Health Care Research and Quality including 18,753 articles found that only 47 (3%) trials included an active control treatment [80]. This approach only contributes to showing the absolute benefit of an MBI and fails to demonstrate if that MBI has more value than clinical attention alone. Based on our previous findings, accounting for therapeutic contact with clinicians is important in this phase [38]. Moreover, guidance from clinicians embedded in web-based interventions has an impact on outcomes [81]. Hence, attention control is relevant in highlighting the benefits of MBI. Moreover, ACGs may help mitigate the lack of oversight on usual care by standardizing the information received by people in the control groups [82]. Finally, although it is admitted that double blinding is complex to implement in studies with psychosocial interventions, ACGs may contribute to blinding to a certain extent [82]. Thus, an attention control approach is used in this study. Although the web-based standardized session needs to be credible and includes a video recording from a clinician, it was carefully developed so that it does not include any active ingredient [82,83].

### Treatment Conditions

Usual care includes a booklet with brief instructions on postoperative care and surveillance of complications provided during hospitalization, and a general assessment and some instructions regarding medication intake and safe return to activities as needed at the time of the follow-up appointment. In addition to usual follow-up care, in the first week of enrollment, the ACG will have access to a 15-minute standardized educational web-based session on persistent postsurgical pain, how pain and stress may interact, and their potential impact on recovery. Brief instructions on self-care (eg, selecting activities that bring a sense of pleasure or mastery) and helpful self-statements (eg, “I will overcome this pain” and “this pain will not last”) will also be provided. In addition, three weekly reminders will be sent to encourage participants to review the web-based session.

Patients assigned to the EG will receive a brief web-based MBCT intervention adapted from the brief four-session clinical manual on MBCT for chronic pain by day [84]. Four 15- to 30-minute sessions are planned (Textbox 1). Each session is structured as follows: an introductory video of the clinician, mindfulness exercise audio recording, wrap-up and weekly homework, or maintenance instructions from the clinician.

**Outline of the four brief mindfulness-based cognitive therapy sessions.**

**Sessions’ Main Themes and Mindfulness Strategies**

Session 1: persistent postoperative pain

- Body scan (20 minutes)

Session 2: stepping out of automatic thoughts

- Mindful breathing (10 minutes)
- 3-minute breathing space

Session 3: acceptance and self-care activities

- Mindful breathing (10 minutes)

Session 4: wrap-up and maintenance plan

- 3-minute breathing space
- Body scan (20 minutes)

The first session will first focus on providing feedback regarding persistent postsurgical pain, the role played by the brain in pain perception, and the relationships between thoughts, feelings, and behaviors. It will end with teaching one mindfulness strategy, that is, body scan, or bringing detailed awareness to each body part and bodily sensation, which is considered an accessible introductory exercise to mindfulness meditation [47]. The second session will focus on pain-related automatic thoughts and teaching the second mindfulness strategy, that is, mindful breathing or focusing attention on breathing—the inhale and the exhale. Moreover, a brief portable 3-minute breathing space strategy will also be presented. The third session will focus on acceptance and self-care strategies and practicing mindful breathing. The fourth session consists of providing feedback and reminders about cognitive reactions to pain and suggesting a maintenance plan along with an opportunity to practice the strategies. The participants will be asked to practice guided meditation (audio recording) 5 days a week for a total of 4 weeks: beginning with body scan in week 1, mindful breathing and the 3-minute breathing space in week 2, and participants may choose either practice for weeks 3 and 4. Participants will also receive a booklet with information about persistent postsurgical pain, an overview of the 4-week program, and a description of the strategies. Participants in the EG will receive reminders as needed, that is, if they do not view the weekly session at all.

The intervention will start upon follow-up with the cardiac surgeon (usually approximately 2-4 weeks after surgery). This time frame will allow participants to recover from surgery, return to their routine at home, and experience the challenges of managing persistent pain in their home and daily activities. The intervention will end approximately 6-8 weeks after surgery but before pain is considered chronic (ie, 3 months postsurgery). At the time of enrollment, participants will receive education via telephone and email on how to access and implement the brief web-based MBCT intervention or the attention control intervention. Participants will be informed that weblinks (Qualtrics) for each weekly session will be sent via SMS text messaging or email. The link will redirect participants to a Qualtrics page where video and audio recordings are posted. Qualtrics allows viewing videos from any type of device, for example, a computer, tablet, or smartphone. Participants’ progress and adherence will be monitored on web using Qualtrics.

### Data Collection

As mentioned, measures will be taken via a telephone interview using Qualtrics for data entry. Given the pilot nature of this study and the timeline of the CPSP trajectory [85], all measures, except for acceptability and feasibility of the intervention, will be taken before the intervention (T0), after the intervention (T1), 3 months after surgery (T2), and 6 months after surgery (T3) for both groups [85]. Usual sociodemographic variables (ie, age, sex, civil status, living conditions, education level, and employment status) will be assessed. Relevant medico-surgical information during hospitalization will be collected: type of surgery, presence of postoperative complications, length of hospital stay, and current involvement in a rehabilitation program. The presence of chronic pain before surgery will also be documented. Considering established relations between pain, anxiety, and depression [86-88], measures of anxiety and depression will be taken with the Patient Health Questionnaire-4 for depression and anxiety [89] at all time points. The validity and reliability of the Patient Health Questionnaire-4 has been well established [89-91].

### Intervention Acceptability and Feasibility

An assessment of the acceptability of the intervention will be conducted at the end of the intervention (T1) using a questionnaire. Furthermore, 30-minute semistructured individual interviews (telephone or videoconference) will then be conducted by the RA. The intervention components will be rated in terms of four attributes: (1) appropriateness in helping patients manage pain, (2) effectiveness in promoting pain self-management, (3) suitability, and (4) willingness to adhere with the use of the treatment acceptability and preference measure [92,93]. The ratings refer to a 5-point scale ranging from 0 (not at all) to 4 (very much). A total scale score between 0 and 4 was obtained as the mean of the four items to reflect perceived intervention acceptability. The four items demonstrated internal consistency reliability (α>.80) [93] and were validated in this population when used to assess the first module of the intervention [39]. The patients’ rating of each component will be used to solicit feedback on the acceptability of the intervention and on the need for further modifications during interviews. The interviews will be digitally recorded and transcribed by a trained RA. Regarding feasibility, in addition to refusal, dropout, and withdrawal rates, field notes will be taken during testing regarding various criteria: fidelity in terms of planned mode, dose, timing, and activities, but material resources and context [92]. Percentages of participants completing the sessions according to the planned schedule and the number of times the sessions were accessed by participants will be assessed through Qualtrics monitoring.

### Pain

Pain intensity is a global indicator of pain. It will be assessed using a numerical rating scale (0-10), with the anchors being *no pain at all* (score=0) and *worst possible pain* (score=10). This type of scale is recognized for its reliability, validity, and sensitivity in various clienteles and settings, including patients undergoing cardiac surgery [79,94,95]. Four different measures of pain intensity will be taken: (1) average pain upon movement in the past 7 days, (2) worst pain upon movement in the past 7 days, (3) present pain upon movement, and (4) present pain at rest.

### Pain Interference With Daily Activities

As suggested by the Initiative on Methods, Measurement, and Pain Assessment in Clinical Trials group with regard to pain core domains in clinical trials [79,96], the impact of pain on various aspects of daily living will be assessed with interference items of the Brief Pain Inventory [97]. The Brief Pain Inventory has been successfully validated in patients undergoing cardiac surgery in the context of both acute and chronic pain and intervention research [66,98,99]. It includes 7 items and evaluates the impact of pain on general activity, mood, walking, work, relationships, sleep, and enjoyment of life. Some items were added in earlier studies to measure postoperative pain-related interference with appetite, concentration, and breathing or coughing [66,99]. Each item represents a subscale and can be scored (range: 0-10) and analyzed individually, with the anchors being *does not interfere* (score=0) and *completely interferes* (score=10). The total interference score can also be calculated by considering the sum of all items.

### Opioid Use and Misuse

Analgesic medication intake will be documented at all time points. If some participants report that they currently take opioids, they will complete the Opioid Compliance Checklist [100] on opioid misuse. This instrument is a brief self-report measure comprising eight items with a yes or no answer. A greater number of positive answers reflects an increased risk of current and future opioid misuse. Its validity and reliability in detecting drug-related behaviors have been well established [101].

### Mindfulness, Chronic Pain Acceptance, and Pain-Related Catastrophic Thoughts

Given that the intervention is meant to increase mindfulness and the associations between mindfulness measures, pain acceptance, and pain-related catastrophic thoughts [21,61,62], mindfulness will be assessed. The Cognitive and Affective Mindfulness Scale-Revised [102] will be used to assess mindfulness. This 12-item scale captures the broad concept of mindfulness according to four domains (attention, present focus, awareness, and acceptance or nonjudgement) without being specific to any type of meditation or strategy. Each item is rated on a 4-point scale with the end points *rarely or not at all* (score=1) and *almost always* (score=4). The total score is calculated by taking the sum of the items. The Cognitive and Affective Mindfulness Scale-Revised has demonstrated good consistency for the overall score [102] and has been validated with MBI in patients with persistent pain [50]. The Chronic Pain Acceptance Questionnaire-8 [103] is an 8-item questionnaire measuring chronic pain acceptance. This scale comprises two subscales: the degree to which patients engage in daily living activities regardless of pain (4 items) and willingness to experience pain (4 items). It has demonstrated good consistency, and its sensitivity to therapeutic changes has been validated [104]. The Pain Catastrophizing Scale [105] will be used to assess patients’ pain-related catastrophic thoughts. It includes 13 items divided into three subscales: rumination (4 items), magnification (3 items), and helplessness (6 items). Each item is rated on a 5-point scale with the end points *not at all* (score=0) and *all the time* (score=4). The total score and scores for each subscale can be calculated by taking the sum of the items. The Pain Catastrophizing Scale has demonstrated an excellent internal consistency [105], and its sensitivity to psychosocial interventions for chronic pain has been established [106,107].

### Statistical Analysis

With regard to the first aim, the intervention acceptability scores will be summarized using descriptive statistics. Qualitative data obtained from individual interviews will be analyzed [108] using NVivo software. A preliminary generation of codes or categories based on acceptability attributes from the treatment acceptability and preference measure (ie, appropriateness, effectiveness, suitability, and willingness to adhere) will be used. Double coding will be conducted by the PI and RA. The results will be compared and discussed until a consensus is reached. Frequency counts will also be used to confirm the emergence of themes. Triangulation of both qualitative and quantitative data will be used to develop a more comprehensive understanding of the acceptability of the intervention.

Regarding the preliminary effects of the intervention, the protocol will favor an intention-to-treat approach for the analysis of results, thus involving all patients who were randomly assigned. Participants’ flow will be reported according to the Consolidated Standards of Reporting Trials guidelines for psychological interventions [109]. The statistical analysis will be mostly descriptive (mean and SD for continuous outcomes and frequency and proportion for categorical outcomes) with 95% CIs when appropriate. Pain intensity, pain interference, opioid use, mindfulness, pain acceptance, pain-related catastrophic thoughts, and depression or anxiety scores will be summarized using descriptive statistics presented per group at each time point. Furthermore, treatment effects will be estimated and presented with 95% CIs at each time point. Between-group differences will be assessed by fitting linear mixed models for each outcome: pain intensity, pain interference, opioid use, mindfulness, pain acceptance, pain-related catastrophic thoughts, and anxiety or depression. Outcome variables will be regressed on the intervention group (EG vs ACG) and time (preintervention, postintervention, and 3 and 6 months after surgery) after covarying baseline values to perform statistical matching on prerandomized values, thus ensuring that comparisons by treatment group are independent of baseline differences [110]. Of primary interest is the impact of the intervention on the prevalence and severity of CPSP (ie, pain intensity, pain interference, and opioid use) at 3 and 6 months. However, these analyses will be undertaken primarily for illustrative purposes, as the study is not powered to show statistical significance. An α level of significance of .05 will be used for all analyses.

## Results

Recruitment began in June 2021. Although several clinical settings expressed a great interest for the study and type of intervention, recruitment is not progressing as expected as passive recruitment strategies were used because of the current pandemic. The decrease of elective procedures such as cardiac surgeries during surge of COVID-19 cases is also a considerable barrier. The recruitment process is constantly monitored by the research team, discussed with clinical partners, and updated according to the latest data and restrictions related to COVID-19. Data collection is expected to be completed by March 2022.

## Discussion

### Principal Implications

Pain after cardiac surgery is the most common symptom. However, multiple challenges still exist regarding its management [111]. The impact of unrelieved pain after cardiac surgery can be long lasting and can even influence the trajectory of cardiovascular disease [10,11]. For a few years now, a multimodal approach has been the gold standard for postoperative pain management [112]. Our previous work has demonstrated the potential to influence pain management behavior and postoperative recovery in the first days after surgery with a brief intervention if content targets specific psychosocial risk factors for CPSP. Most importantly, the intervention approach targets psychological factors that play an important role in the transition from acute to chronic postoperative pain. A logical next step in this research regarding the prevention of CPSP is to explore the potential impact of such interventions on pain after discharge from the hospital when patients are more isolated and need to self-manage their pain. In addition to our preliminary work in the acute phase, an intervention tackling pain in the different phases of the perioperative continuum could be proposed. The ultimate goal is to prevent the development of chronic pain and associated disability and opioid misuse.

### Significance of Intended Outcomes

Achieving these aims is significant because they directly target the trajectory of CPSP, a leading cause of disability and opioid misuse. This approach is innovative because it promotes pain self-management through the enhancement of individual protective factors and modulation of individual risk factors. Further MBI for pain is fairly recent and has not been examined for its potential role in preventing chronic pain. Of note, preventing the transition to chronic pain and reliance on opioids while promoting patient engagement and care accessibility is closely in line with the National Institute of Health’s National Pain Strategy [23] and Helping to End Addiction Long-term initiative [113]. Finally, the lack of continuity of care has emerged as a barrier to the prevention of CPSP [111]. With the proposed approach, patients can access the intervention at their convenience and according to their needs but also gain autonomy through a self-management approach. Indeed, the use of interactive technologies helps address the challenges of continuity of care between the different phases, as well as accessibility to health education and evidence-based quality pain care, which is a current national concern [23].

### Conclusions

This funded pilot RCT will examine the acceptability, feasibility, and preliminary efficacy of a web-based MBI for the prevention of CPSP during the recovery period after discharge from hospital. The results of this study will inform current clinical guidelines promoting a multimodal approach to pain management and continuity of care. If successful, the intervention could be expanded to numerous surgical populations at risk of developing CPSP, such as orthopedic surgical patients and other patients at risk for chronic pain, persons with work-related injuries, or persons living with a chronic disease. After further evaluation, a brief and cost-effective intervention could be introduced to hospitals, rehabilitation programs, and other primary care settings to support clinicians and patients in their partnership for the prevention of CPSP. The high potential of integration of the intervention in established care programs, such as rehabilitation programs, is promising for its uptake and sustainability.

## Appendix

Multimedia Appendix 1 Peer-reviewer report from Florida State University's Council on Research & Creativity.

## Abbreviations

ACG: attention control group
CPSP: chronic postsurgical pain
EG: experimental group
MBCT: mindfulness-based cognitive therapy
MBI: mindfulness-based intervention
PI: principal investigator
RA: research assistant
RCT: randomized controlled trial
