# Supplementary material for: Web- and Mindfulness-Based Intervention to Prevent Chronic Pain After Cardiac Surgery: Protocol for a Pilot Randomized Controlled Trial
Source: JMIR Res Protoc. 2021 Aug 30;10(8):e30951. doi: 10.2196/30951 (PMC8438614; doi:10.2196/30951)
Supplement: Multimedia Appendix 1 [file resprot_v10i8e30951_app1.pdf]

CRC Grant Proposal Review Summary

**CRC Program**	Planning Grant
**Project Title**	Feasibility and preliminary effects of a tailored mindfulness-based intervention to prevent chronic pain during rehabilitation after cardiac surgery
**PI Name**	geraldine martorella
**Department**	Nursing Department (NURSING)
**Scores**	1 | 2 | 3 Scores range from 1-9 with 1 being the best possible score
**Average**	2
**Trimmed Mean**	2
**Amount Requested**	$18,845

**Score**	1
**Strength**	This proposal is fantastic, extremely detailed and clearly important in its impact for public health. I also appreciate the very detailed explanation of how this work differs from previous projects and why it is unique and important for the PI's overall research agenda and patient outcomes.
**Weakness**	none
**Revisions Requested?**	No

**Score**	2

**Strength**	Strong introduction, orienting the reader to the literature in the field. Figure 1 was helpful in understanding the conceptual framework.<br>Clear description of the methodology.<br>Appropriate external funding plan.<br>This line of work (for which this specific project would serve as a pilot) is potentially high impact in the long run.
**Weakness**	No major weaknesses noted.
**Revisions Requested?**	⊘ No

**Score**	3
**Strength**	This proposal tackles an important and timely issue, pain self-management in the rehabilitation phase following cardiac surgery, that has the potential to attract external funding.<br>The intervention proposed utilizes modern technology that lends itself 2 high levels of scalability and therefore potential widespread use in the target population.<br>The proposal Considered favorably in the spring has been modified to be sensitive to covered restrictions.<br>Establishing the acceptability of an intervention is an important part of its development.

**Weakness**	+ There appears to be a problem in the study plan. Specifically, subjects will be recruited four to six weeks post operation when they seek rehabilitative services. The intervention itself is 4 weeks. Assessments will be taken at baselineT0 , postintervention (t1), three months post operation (t2) and at six months post operation (t3). It is likely that some subjects will be recruited at six weeks post operation and may only begin the intervention in week seven or eight post operation. In such cases the interval between t1 and t2 will likely only be a matter of days. This raises questions about potential carryover effects from one assessment to the next. For those recruited promptly at four weeks post operation, the interval would be longer and perhaps acceptable.<br>+ There were also some minor but distracting issues in the writing. For example, “The RA who will be responsible of the entire data collection.” An RA is responsible FOR a data collection.<br>+ Finally, funds are sought to underwrite the publication of two open access articles. It is difficult to imagine that these pilot data will support the publication of two articles. This is particularly the case given the timeline for the data collection. Recruitment and data collection will continue until the last month of the grant.
**Revisions Requested?**	⊘ No

© Florida State University | Tallahassee, FL 32306 | 850-644-9694
